# Temporal trends in respiratory syncytial virus-related infant mortality and hospitalizations in the United States

**DOI:** 10.1038/s41390-026-05001-8

**Published:** 2026-04-20

**Authors:** Arya Patel, Ramesh Vidavalur, Chintan K. Gandhi

**Affiliations:** 1 Divison of Neonatal-Perinatal Medicine, Department of Pediatrics, The Pennsylvania State College of Medicine, Hershey, PA, USA.; 2 Division of Neonatal-Perinatal Medicine, Department of Pediatrics, Cayuga Medical Center/Weill Cornell Medicine, Ithaca, NY, USA.

## Abstract

**BACKGROUND:**

Respiratory syncytial virus (RSV) is a leading cause of infant mortality and morbidity, yet national trends in RSV outcomes, particularly during the COVID-19 era, are not well defined. This study evaluated temporal patterns in RSV-related infant mortality and hospitalizations across the United States.

**METHODS:**

We analyzed RSV-associated infant mortality data from 2007–2022 using the CDC WONDER database and hospitalization rates from 2018–2025 using the hospitalization surveillance network (RSV-NET). Infant mortality and hospitalization patterns were assessed by season, race/ethnicity, and birth weight.

**RESULTS:**

Over 16 years, RSV-related infant mortality remained relatively stable, with no statistically significant year-to-year variation. Mortality rates were disproportionately higher among Black or African American infants and those with low birth weight. Seasonal hospitalization patterns varied substantially: the 2020–2021 RSV season recorded the lowest hospitalization rate, likely reflecting widespread COVID-19 mitigation measures, whereas the 2022–2023 season recorded the highest rate since 2018.

**CONCLUSION:**

Although infant RSV mortality has remained stable, persistent disparities exist, and hospitalization rates show substantial seasonal fluctuations. These findings provide critical baseline data for assessing the impact of emerging RSV immunization strategies and highlight the need for targeted strategies to reduce persistent inequities in infant outcomes in the post–COVID-19 era.

## INTRODUCTION

Respiratory syncytial virus (RSV) remains a leading cause of morbidity and mortality in children with greatest burden during the first year of life.^1,2^ In many developed countries, including the United States (US), RSV is the most common cause of lower respiratory tract infections, hospitalizations, and mortality among children under the age of five.^3,4^ Recent years have brought significant advances in developing novel prophylactic strategies to protect infants during their first RSV season. These include a monoclonal antibody targeting the RSV fusion (F) protein for infants younger than 8 months of age,^2,5,6^ and a bivalent RSV prefusion F protein vaccine administered to pregnant individuals.^7^ Clinical trials and post-licensure data suggest these interventions can reduce severe RSV disease by approximately 70–90%.^8,9^ Despite these achievements, RSV immunization uptake remains suboptimal around 50–60%,^10^ and about 8% of immunized children still require intensive care, with the greatest impact observed in otherwise healthy newborns.^11,12^

The post-COVID-19 pandemic era has also altered seasonal circulation patterns of respiratory viruses, including RSV.^13^ Current national RSV-related mortality estimates are largely based on earlier decades that covered the cohort periods 1990–1999,^14^ 1997–2009,^15^ and 1998–2018.^16^ Although a few studies have examined the shifts in epidemiology and severity of RSV during COVID-19 pandemic,^13,17^ none have assessed the impact of the COVID-19 pandemic on RSV-related hospitalizations or mortality in US infants at the national level. A study by Irving et al. reported high overall infant RSV immunization coverage (72%) in the Vaccine Safety Datalink during the 2023–2024 season, achieved through either maternal vaccination or infant immunization. However, substantial racial and ethnic disparities were observed, with lower coverage among infants of Black and Middle Eastern and North African mothers compared with Asian mothers, underscoring an urgent need to address access gaps.^18^ Consistent with these findings, a recent CDC report noted marked state-level variability in infant RSV immunization coverage during the 2023–2024 season, ranging from an estimated 11%–53%.^9,19^

These data gaps reinforce the critical need for a comprehensive and updated assessment of RSV epidemiology in the current era. This study aims to address this gap by analyzing RSV-related infant mortality and hospitalization trends in the US from 2007 to 2022. Updated burden estimates are essential to characterize RSV epidemiology, track annual changes, and understand shifts in demographic profiles, assess the influence of evolving healthcare practices, and public health interventions. Building on previous research, our primary objective is to identify trends in RSV-related mortality, describe RSV-associated hospitalization burden and to evaluate differences in hospitalization rates among infants across the US.

## METHODS

In this cross-sectional study, we analyzed mortality data from the Centers for Disease Control and Prevention Wide-Ranging Online Data for Epidemiologic Research (CDC WONDER) Linked Birth/Infant Death database, covering the period from January 1, 2007, to December 31, 2022.^20^ This period represents the most recent years with finalized mortality data available at the time of analysis. The CDC WONDER database is a crucial information resource available to academic researchers and the public for examining multiple causes of infant mortality and temporal trends. These data, derived from U.S. death certificates, include the number of cause-specific deaths and crude death rates. Since 1999, causes of death have been classified using the International Classification of Diseases, Tenth Revision (ICD-10) codes. The database allows analyses stratified by key variables, including county of the mother’s residence, infant age, underlying cause of death, sex, birth weight, birth plurality, birth order, gestational age at birth, and maternal race and ethnicity. Data use restrictions prohibit the publication or presentation of death counts of nine or fewer, or death rates calculated from counts of nine or fewer.

Data on RSV-associated infant hospitalizations were obtained from the Respiratory Syncytial Virus Hospitalization Surveillance Network (RSV-NET) for the 2018–2025 RSV seasons.^21^ RSV-NET conducts population-based surveillance of laboratory-confirmed RSV hospitalizations among children and adults in select counties across 12 states at the time of analysis. This study utilized publicly available, de-identified, aggregated data and was exempt from institutional review board (IRB) oversight under the Federal Common Rule, which allows exemption for public health surveillance activities related to disease trend monitoring. The Cayuga Medical Center IRB classified this research as exempt, determining it did not involve human participants and waived the requirement for informed consent. The study adheres to the Strengthening the Reporting of Observational Studies in Epidemiology (STROBE) guidelines.

### RSV-attributable infant mortality

Infant deaths from RSV were identified using International Statistical Classification of Diseases, Tenth Revision (ICD-10) codes: J12.1 (RSV pneumonia), J20.5 (RSV bronchitis), and J21.0 (RSV bronchiolitis). The ICD-10 code B97.4 (“Respiratory syncytial virus as the cause of diseases classified elsewhere”) is excluded from the CDC WONDER cause of death dataset by the National Center for Health Statistics and was not included in our analysis (see Supplemental Information). Mortality data were stratified by race (four categories), sex, and year of death. Maternal race was categorized using CDC bridged race classifications (American Indian/Alaska Native, Asian/Pacific Islander, Black, White). Due to CDC suppression rules limiting disclosure of small cell sizes, results are presented only for race groups meeting reporting thresholds.

### RSV-associated infant hospitalization rates

Hospitalization data for laboratory-confirmed, RSV cases were obtained from RSV-NET across seven surveillance seasons (2018–2025). RSV-NET, a collaboration among the CDC, the Emerging Infections Program Network, and the Council of State and Territorial Epidemiologists tracks laboratory-confirmed, RSV-associated hospitalizations in infants (see Supplementary Information). All temporal analyses were conducted using CDC-defined epidemiologic weeks, consistent with RSV-NET surveillance reporting.

To account for temporal dependencies and unmeasured site-level differences, the CDC applies a hierarchical Bayesian modeling approach, specifically, a conditionally autoregressive random effects (CAR-RE) model. For the 2018–2019 and 2019–2020 RSV seasons, data were limited to October through April, reflecting typical pre–COVID-19 RSV seasonality. Beginning with the 2020 season, data were collected year-round (October through September of the following year). Because the analysis was conducted in June 2025, hospitalization rates were included through May 2025. Rates were extracted for infants aged 0–12 months, with subgroup analyses performed for infants aged 0–6 months and 6–12 months. RSV-NET hospitalization data include age-, sex-, and race-stratified information at the aggregate level. However, when analyses were restricted to infants aged <1 year, race-specific hospitalization data were not publicly accessible at the level of granularity required for subgroup analyses at the time of this study. As a result, hospitalization analyses in infants were limited to age- and season-based comparisons.

### Statistical analysis

We calculated summary estimates for RSV-associated infant mortality rates (IMR, per 1 million live births) and hospitalization rates (per 100,000 population). RSV-attributable infant deaths (ages 0–364 days) were analyzed by sex, birth weight, and race. Risk ratios were calculated with Poisson derived 95% confidence intervals (CIs) using MedCalc Software 23.4.4 Version.

Trends in RSV-IMR were evaluated using the National Cancer Institute’s Joinpoint Regression Program (version 5.3.0).^22^ Joinpoint regression is a piecewise linear regression method that identifies inflection points (“joinpoints”) where statistically significant changes in trend occur. For each time segment, we calculated the annual percent change (APC), and for the entire study period (2007–2022), we computed the average annual percent change (AAPC) as a weighted mean of the APCs, with weights based on the segment lengths. A two-sided *p*-value < 0.05 was considered statistically significant. For APC, a *p*-value < 0.05 indicated a statistically significant trend, whereas a 95% CI that included zero or a *p*-value > 0.05 indicated no significant change in trend. The empirical quantile method was used to calculate 95% CIs for both APC and AAPC estimates. We performed sensitivity analyses excluding mortality data from 2022 to assess the robustness of observed temporal trends considering the apparent increase in mortality during that year.

Infant hospitalization rates were determined by dividing the number of laboratory-confirmed RSV hospitalizations among infants by the age-specific mid-year population estimates in each surveillance area. We extracted unadjusted, age-specific (0–364 days) hospitalization data from 12 states (California, Colorado, Connecticut, Georgia, Maryland, Michigan, Minnesota, New Mexico, New York, Oregon, Tennessee, and Utah) to assess trends from 2018 through 2025. Descriptive statistics and the Kruskal-Wallis test were applied to compare mean hospitalization rates across RSV seasons.

## RESULTS

**Fig. 1:**
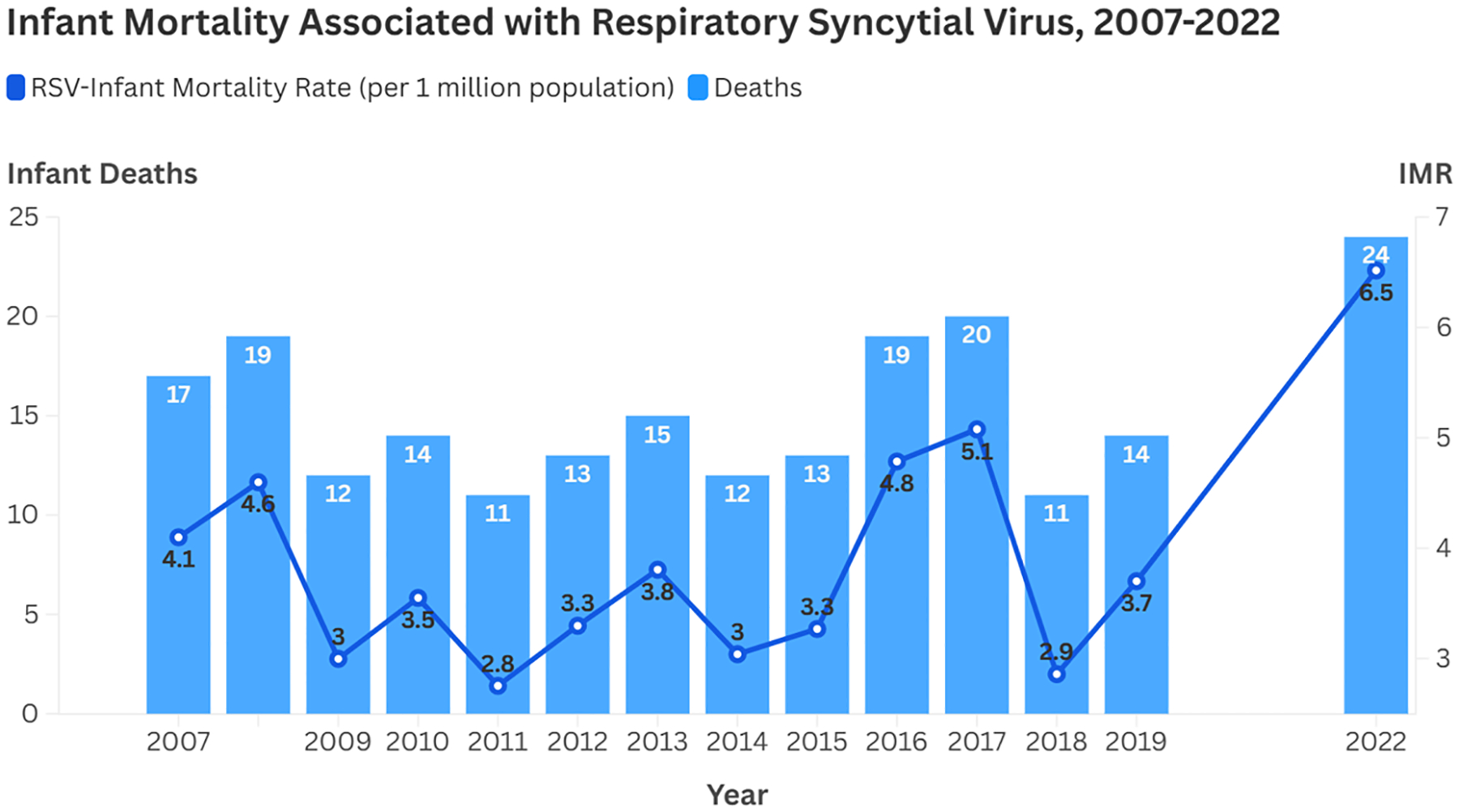
Respiratory syncytial virus (RSV) infant mortality rates, 2007–2022. Secondary y-axis represents mortality rates per 1 million live births.

Between 2007 and 2022, approximately 62.7 million live births occurred in the United States. Of these, an estimated 231 infant deaths (95% CI: 202–262) were attributed to RSV, corresponding to a mean RSV infant mortality rate (RSV-IMR) of 3.8 deaths per 1 million live births (95% CI: 3.2–4.1) (Fig. 1). The annual RSV-IMR increased from 4.1 per 1 million in 2007 to 6.5 in 2022. Despite this apparent increase, Joinpoint regression analysis revealed no statistically significant trend over the 16-year period (APC, 2.0%; 95% CI, −3.4–7.2; *p* = 0.36). In sensitivity analyses excluding 2022 mortality as an outlier, no statistically significant trend changes were observed (APC, 0.3%; 95% CI, −4.2–5.2; *p* = 0.86) (Supplementary Fig. S1). Mortality data for 2020 and 2021 were unavailable due to data suppression policies, as fewer than 10 deaths were recorded each year. As a result, mortality estimates for the pandemic period could not be reliably interpreted, and trends are presented through 2019 and again in 2022.

Univariate analysis stratified by sex, birth weight, and race indicated a lower, non-statistically significant risk of RSV-related mortality among female infants (*p* = 0.08) and a significantly higher risk among infants with low birth weight and those identified as Black (*p* < 0.001) (Table 1).

**Fig. 2:**
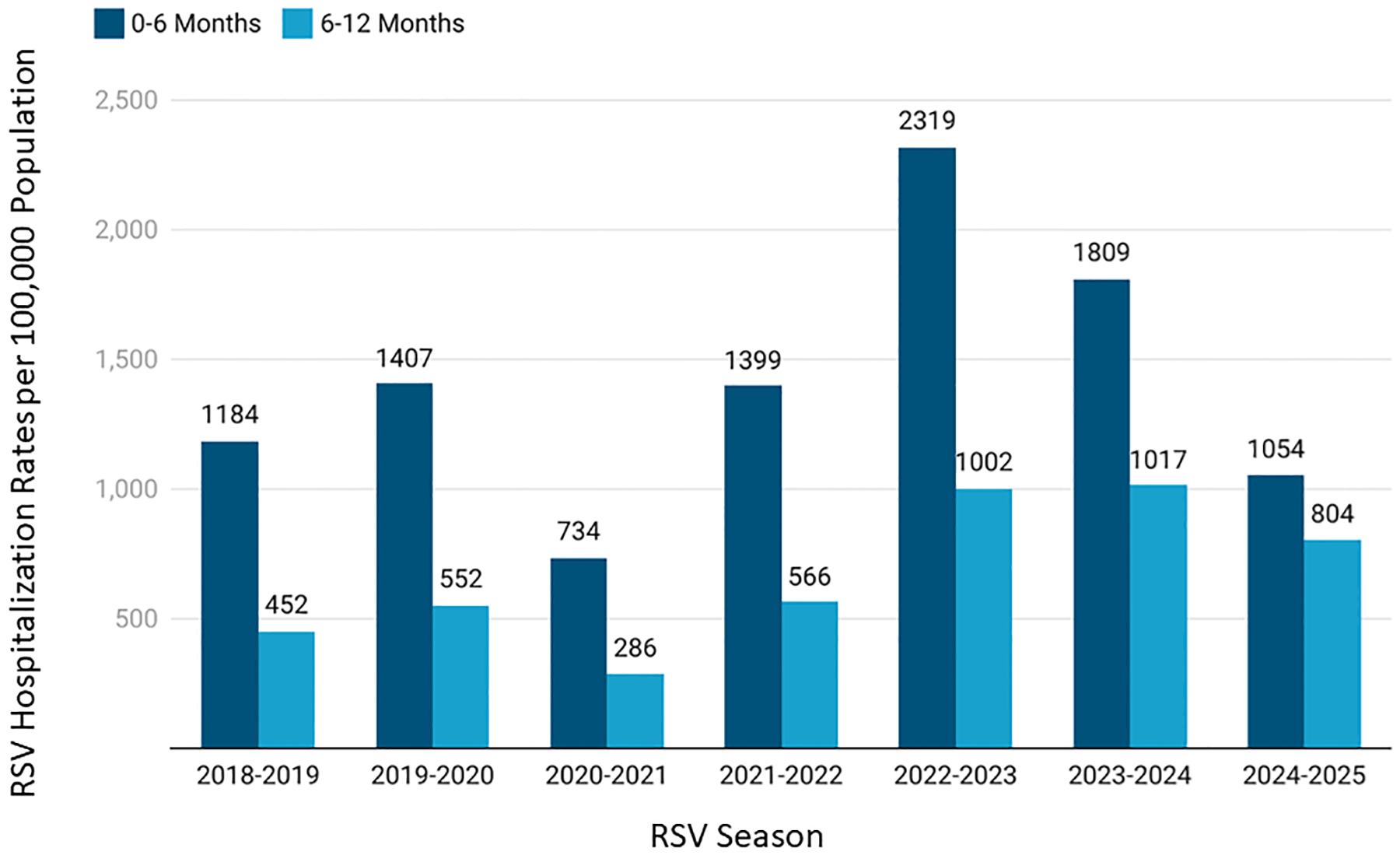
Cumulative respiratory syncytial virus (RSV) hospitalization rates by age group 0–6 months and 6–12 months. Hospitalization rates are presented per 100,000 age-specific population.

Cumulative RSV hospitalization rates per 100,000 population were consistently highest among infants aged 0–6 months, increasing from 1,184 in 2018–2019 to a peak of 2319 in 2022–2023, before declining to 1054 in 2024–2025. Similar trends were observed among infants aged 6–12 months, although rates were substantially lower, rising from 452 in 2018–2019 to a peak of 1017 in 2023–2024, and then declining to 804 in 2024–2025 (Fig. 2). Overall, among all infants aged 0–12 months, hospitalization rates declined from 818 in 2018–2019 to 510 in 2020–2021, rebounded sharply to 1661 in 2022–2023, and subsequently decreased to 1413 in 2023–2024 and 929 in 2024–2025.

RSV-associated hospitalization rates across 12 US states, from 2018 to 2019 through 2024– 2025 seasons, demonstrated considerable year-to-year fluctuations and marked variability across the states (Supplementary Fig. S2). The mean RSV-associated hospitalization rate in the 2018–2019 season was 1001 (95% CI: 720–1282) per 100,000 infants, compared with 923 (95% CI: 627–1219) per 100,000 infants in 2024–2025 season, a difference that was not statistically significant (*p* = 0.68). Over the same interval, hospitalization rates decreased in 9 of the 12 states and increased in Colorado, Georgia, and Maryland.

**Fig. 3:**
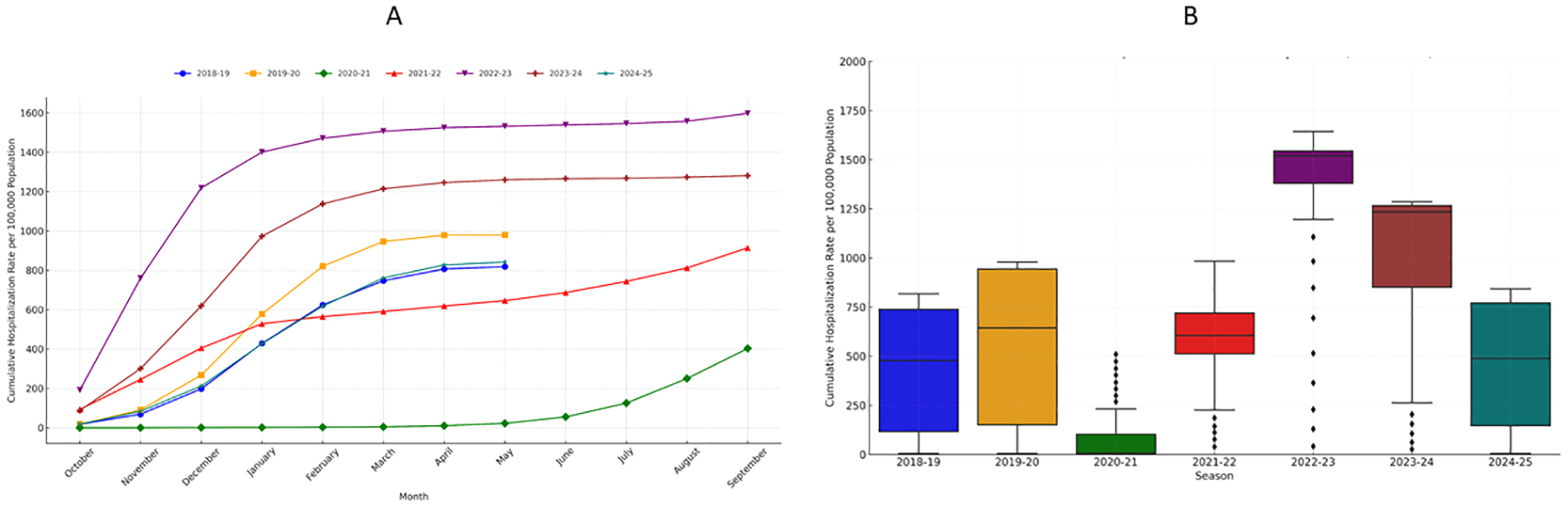
Cumulative respiratory syncytial virus (RSV) hospitalization rates in infants, aggregated across all participating RSV-NET, 2018–2025. **a** Cumulative hospitalization rate by calendar month within each surveillance season. Surveillance data was available for October to April in 2018–2019 and 2019–2020 seasons, and then year-round since October 2020. **b** Overall cumulative seasonal hospitalization rate for each surveillance season, 2018–2025.

Figure 3a illustrates the cumulative RSV-associated hospitalization rate among infants by calendar month within the seven surveillance seasons from 2018–2019 to 2024–2025, aggregated across all participating RSV-NET sites. Figure 3b presents the overall cumulative seasonal hospitalization rate for each surveillance season. Rates varied significantly by season. The highest cumulative rate was recorded in the 2022–2023 season (mean: 1335; 95% CI: 1226–1445), followed by 2021–2022 (mean: 580; 95% CI: 519–641) and 2019–2020 (mean: 558; 95% CI: 424–693). The 2018–2019 season averaged 437 (95% CI: 328–545) per 100,000 population. In contrast, the 2020–2021 season, coinciding with extensive COVID-19 mitigation measures, had significantly lower rates (mean: 78; 95% CI: 42–113). Statistical testing using the Kruskal-Wallis method confirmed significant differences in hospitalization rates across seasons (*H* = 186.49, *p* < 0.001), highlighting the variable burden of RSV during this period.

## DISCUSSION

This study assessed temporal trends in RSV-associated infant mortality from 2007 to 2022 and hospitalization rates from 2018 to 2025 in the United States. Over the 16-year period, RSV-related infant mortality remained relatively stable, with no statistically significant year-to-year differences. Mortality rates were higher among Black or African American infants and those born with low birth weight. Across seven RSV seasons, infants aged 0–6 months consistently experienced the highest hospitalization burden, with a pronounced pandemic-related nadir in 2020–2021 followed by a sharp rebound peaking in 2022–2023. Hospitalization trends among infants aged 6–12 months and the overall 0–12-month group followed similar but attenuated patterns, with declines observed in the most recent season. Although the post–COVID-19 resurgence in RSV hospitalizations complicates interpretation, the recent decrease in hospitalization rates may reflect the combined effects of population immunity, evolving viral dynamics, and early uptake of RSV preventive interventions.^23,24^ Continued surveillance will be essential to distinguish these influences and to assess whether sustained reductions in RSV-related hospitalizations occur as prevention strategies become more widely implemented.

A recent study by Reichert et al. reported a statistically significant decline in RSV-related mortality, from 8.1 per 1 million live births in 2008 to 3.4 in 2018.^25^ In contrast, we observed no significant temporal trend but a nonsignificant increase in the annual RSV-IMR from 4.1 per 1 million in 2007 to 6.5 in 2022. This discrepancy likely reflects differences in data sources and case definitions. Reichert et al. used the National Center for Health Statistics (NCHS) dataset and included broader bronchiolitis ICD codes (J21.8: acute bronchiolitis due to other specified organisms; J21.9: acute bronchiolitis, unspecified), whereas our analysis relied on CDC WONDER data and was restricted to RSV-specific codes. Given the overall decline in U.S. infant mortality over the past two decades, inclusion of nonspecific diagnostic codes in the earlier study may have contributed to its observed downward trend. Moreover, the mortality trends in our analysis largely reflect the pre-implementation period of newly approved RSV prevention strategies, including maternal vaccination and infant monoclonal antibody prophylaxis; thus, these findings establish a baseline against which future reductions in RSV-related infant mortality may be evaluated as uptake of these interventions increases.

Our analysis included mortality data through 2022, encompassing the COVID-19 pandemic period. To our knowledge, no prior study has reported RSV mortality during and after the pandemic. Due to confidentiality restrictions in CDC WONDER (suppression of counts <10),^20^ mortality estimates were unavailable for 2020 and 2021. Nevertheless, we speculate that RSV-related mortality during these years was significantly lower because of nonpharmaceutical interventions implemented to curb COVID-19 transmission. This is supported by our own and others’^26–28^ findings of markedly reduced hospitalization rates in the 2020–2021 season, followed by the highest hospitalization rate in 2022–2023. During the rebound, we observed the highest rate of RSV-related mortality (6.5 per 1 million live births) in 2022 since 2007. These findings are consistent with the “immunity debt” hypothesis, whereby reduced RSV exposure during the pandemic may have increased susceptibility and severity in infants once mitigation measures ended.^29,30^ Other potential contributors to the 2022–23 surge may include circulation of a more virulent RSV strain or immune dysregulation following prior COVID-19 infection.^31^

Consistent with prior studies,^32,33^ we observed differences in RSV-related severity based on sex, with a trend towards higher RSV-related mortality in males compared to females. We also observed significantly higher RSV-related mortality in lowbirthweight and Black or African American infants. These findings align with previous research showing an increased risk of severe RSV among infant born with a birthweight less than 1500 g.^34–36^ Racial disparities may intersect with birthweight risk, particularly among African American infants, who experience higher rates of preterm birth and low birthweight.^37^ Although low event numbers precluded multivariable analyses to assess independent effects, this limitation underscores the need for future analyses as additional mortality data accrue. Overall, persistent disparities in RSV burden likely reflect a complex interplay of biological vulnerability and social determinants of health, including structural inequities and access to care.

### Limitations

This study has several limitations. First, it relies on death certificate data, which are subject to potential misclassification of cause of death. Second, the analysis lacked detailed clinical and sociodemographic information, including household income, parental education, employment status, health literacy, insurance coverage, or U.S. nativity. Third, confidentiality restrictions within the CDC WONDER database prevent reporting of suppressed mortality data for disaggregated racial subgroups (Asian or American Indian populations). As a result, aggregated analyses may obscure important disparities in RSV-related infant mortality among specific racial and ethnic groups. In addition, although RSV-NET collects age-, sex-, and race-stratified hospitalization data across pediatric and adult populations, race-specific hospitalization data for infants aged <1 year were not publicly available at sufficient granularity at the time of analysis. As a result, we were unable to assess racial or other sociodemographic disparities in RSV-associated infant hospitalizations comparable to those examined for RSV-attributable mortality using CDC WONDER data. Fourth, although ICD-10 coding for RSV is highly specific and demonstrates strong positive predictive value in hospitalized pediatric populations, variable diagnostic sensitivity in outpatient settings may result in underascertainment and incomplete case capture.^38–41^ Finally, hospitalization findings may not be nationally generalizable, as RSV-NET represents catchment areas from participating states; observed variability across states likely reflects regional heterogeneity rather than national rates. Ongoing expansion to additional sites may improve national representativeness in future years.

Our geographic analysis was limited to a broad overview of trends across selected U.S. states, which may mask important variations at the state and county levels. Geographic granularity is critical, as RSV surveillance practices and public health interventions vary substantially across jurisdictions, shaped by local healthcare policies, resource allocation, and community engagement strategies. RSV-NET continues to expand its geographic coverage and clinical data collection, improving completeness and representativeness over time; by 2025–2026, surveillance encompassed 170 counties across 14 states, capturing approximately 11% of the U.S. population. Although these enhancements strengthen hospitalization estimates, geographic limitations persist and should be considered when interpreting trends. Future research should incorporate finer geographic resolution and disaggregated data by race and region, as well as clinical and sociodemographic factors, to better characterize RSV epidemiology and inform targeted prevention and screening strategies for high-risk populations.

Despite these limitations, our findings, combined with existing RSV burden estimates,^14–16,25^ provide a comprehensive overview of RSV morbidity and mortality in infants during and after the COVID-19 pandemic in the United States. With the recent approval of RSV immune-prophylaxis practices, these results offer valuable baseline information to evaluate intervention effectiveness, identify barriers to equitable uptake, and guide strategies for sustained implementation in real-world settings.

## CONCLUSION

RSV-associated infant mortality in the United States remained stable over the past decade, with no significant year-to-year change in mortality rates. In contrast, RSV-associated hospitalization patterns showed inter-state heterogeneity, with substantially higher rates among infants younger than six months. The earliest post-immunization seasons demonstrated substantial declines in infant hospitalization rates, suggesting a possible emerging impact of RSV prevention products that warrants continued surveillance. These findings establish a national pre-immunization baseline and underscore the need for ongoing monitoring to evaluate long-term trends, equity implications, and the sustained effects of prevention strategies on RSV disease burden.

## Supplementary Material

Supplemental Information

The online version contains supplementary material available at https://doi.org/10.1038/s41390-026-05001-8.

## Figures and Tables

**Table 1.** Study participants characteristics, 2007–22.

	Deaths	Births	RSV IMR*	Rate Ratio (95%CI)	P value
	(n=231)	(n=62,785,519)			
*Sex*					
Female	100	30,665,299	3.3	Ref	
Male	132	32,120,220	4.1	1.3 (0.9, 1.6)	0.08
*Birth weight (gms)*					
<1499	51	901,305	56.6	26.7 (18.8, 37.3)	<.001
1500–2499	58	4,262,143	13.6	6.4 (4.6, 8.8)	<.001
>2500	122	57,622,071	2.1	Ref	
*Race* **					
Black or African American	57	8,403,334	6.8	2.2 (1.6, 3.1)	<.001
White	118	39,321,052	3.0	Ref	
Not Reported	40	10,945,697	3.7	1.2 (0.8, 1.7)	0.28

* Per 1 million births

** Asian and American Indian infant mortality values do not meet confidentiality constraints

RSV – Respiratory syncytial virus, IMR – Infant mortality rate, CI – Confidence interval

## Data Availability

Data will be made available upon reasonable request.

## IMPACT:

- RSV-related infant mortality in the United States has shown both stability and a sharp post-pandemic surge, with persisting disparities by sex, birthweight and race.
- This study integrates temporal, demographic, and geographic patterns to provide the national-level evidence of RSV mortality and hospitalization patterns among infants during COVID-19 era.
- These findings establish essential baseline estimates to evaluate effectiveness of preventive measures, while underscoring the urgency of targeted strategies to reduce disproportionate risks among vulnerable infants.
