# Supplementary material for: Temporal trends in respiratory syncytial virus-related infant mortality and hospitalizations in the United States
Source: Pediatr Res. Author manuscript; Available in PMC 2026 Jul 13. (PMC13359670; doi:10.1038/s41390-026-05001-8)
Supplement: Supplemental Information [file NIHMS2191585-supplement-Supplemental_Information.pdf]

Supplemental Information

Infant mortality attributable to RSV:

It has been recognized that RSV is underreported on death certificates. RSV may lead to death from other causes, such as bacterial pneumonia, cardiac complications, or chronic obstructive pulmonary disease; these RSV-associated deaths may be attributed to these other causes of death on death certificates. Some reasons for underreporting may be that patients aren't always tested for RSV, particularly older adults who are at greatest risk of RSV complications and death, and not all deaths related to RSV occur in the hospital.

Because there is underreporting on death certificates, only counting deaths where RSV was recorded on a death certificate would greatly underestimate RSV's true impact. Modeling strategies are therefore commonly used to estimate total RSV–associated deaths.

In-hospital deaths among those hospitalized with laboratory-confirmed RSV and observed in RSV-NET were adjusted for under-detection of RSV using data on the frequency and sensitivity of RSV testing among those who died in the hospital with illnesses compatible with RSV.

In addition, death certificate data were used to estimate how likely RSV–associated deaths were to occur outside the hospital. Death certificates that had pneumonia or influenza causes (P&I), other respiratory and circulatory causes (R&C), or other non-respiratory, non-circulatory causes of death were also examined.

The proportion of these deaths that occurred while in the hospital was then estimated using data provided by the National Center for Health Statistics (NCHS). Information on the causes of death from RESP-NET was used to determine the mixture of P&I, R&C, and other coded deaths to include in the investigation of death certificate data. Finally, once the proportion of RSV–associated deaths that occurred outside of the hospital was estimated, the death-to-hospitalization ratio was estimated.

Data needed to estimate RSV-associated deaths may lag for up to 2 years after the season ends. When this was not yet available for the season being estimated, adjusted estimates based on values observed in prior seasons were used and estimates updated when contemporaneous data became available.

RSV associated infant hospitalization rates:

RSV-associated hospitalization rates are used to understand trends in virus circulation, estimate disease burden, and respond to outbreaks. However, the rates are unadjusted and do not account for under testing, differing provider or facility testing practices, and diagnostic test sensitivity. The rates presented in the interactive dashboard might underestimate the true burden of RSV-associated hospitalizations in the United States, but the trends by age group and geographic location remain useful for monitoring disease severity.

To estimate national RSV-associated hospital admissions from RSV-NET data, CDC used a hierarchical Bayesian modeling approach, namely, the Conditionally Autoregressive–Random Effects (CAR-RE) model. The CAR-RE model accounts for dependencies across weeks in RSV-

NET reported hospitalizations via conditionally autoregressive (CAR) and site-level random effects (RE) to reflect unmeasured differences among the sites participating in the RSV-NET surveillance system. Additionally, the CAR-RE model approach enables estimation of the uncertainty in the derived national hospitalization counts due to extrapolation from the area covered by RSV-NET surveillance to the entire US population.

Modeled hospitalization counts are adjusted to reflect under-detection using both the estimated probability that an inpatient is tested for RSV infection and the expected sensitivity of the utilized tests. Testing is subject to provider discretion, facility policy, and test availability. The data on testing can lag, so testing data from recent prior years are applied to the current season estimates.

The adjusted RSV-associated hospitalization estimates are then used to estimate additional measures of RSV-associated burden including outpatient visits and deaths via a probabilistic mathematical multiplier model using Monte Carlo simulations.

References:

1. RSV-NET: Respiratory Syncytial Virus Hospitalization Surveillance Network, Centers for Disease Control and Prevention. https://www.cdc.gov/rsv/php/surveillance/rsv-net.html. Accessed on 5/6/2025”.
2. https://wonder.cdc.gov/wonder/help/lbd.html#
3. How CDC Estimates the Burden of RSV in the US: https://www.cdc.gov/rsv/php/surveillance/about-burden-estimates.html

Supplemental Figure S1. Joinpoint Trend Sensitivity Analysis. RSV-Associated Infant Mortality, 2007-2019; 2007-2022

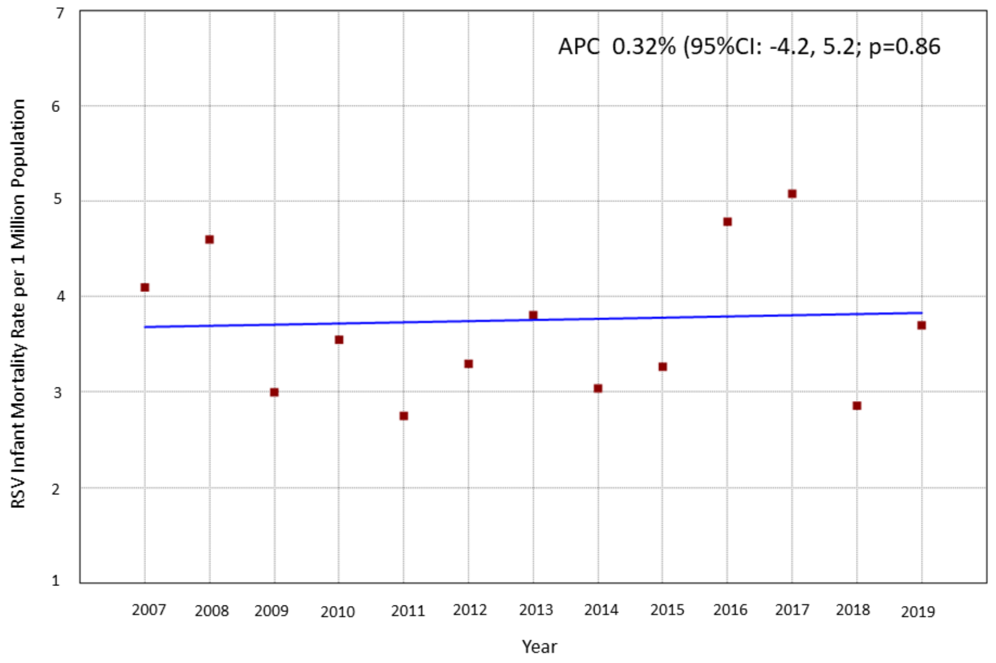

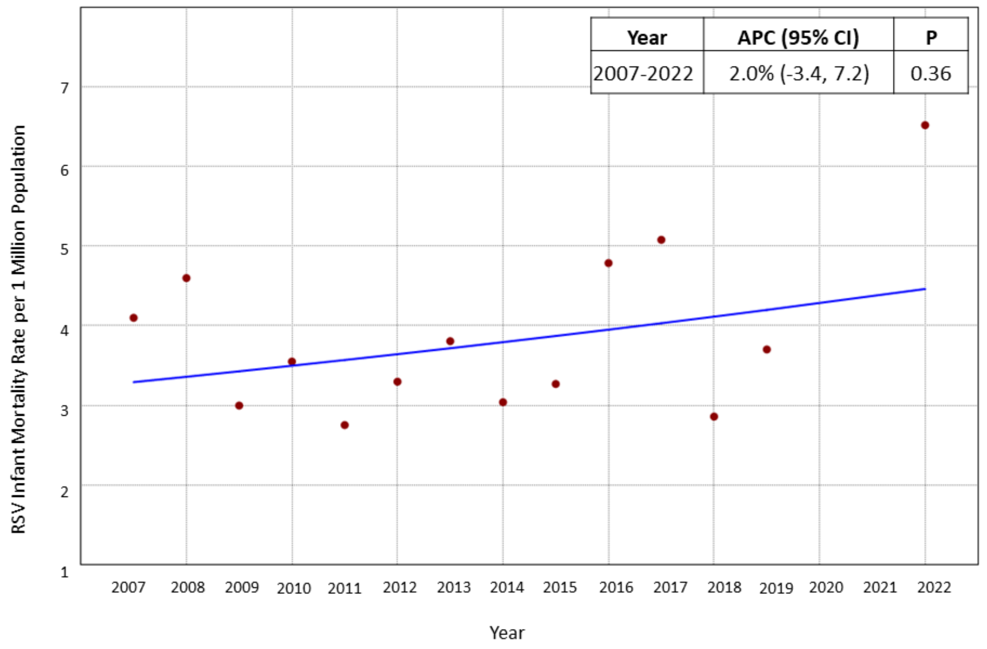

Supplemental Figure S2. RSV Hospitalization Trends by States, 2018-2025

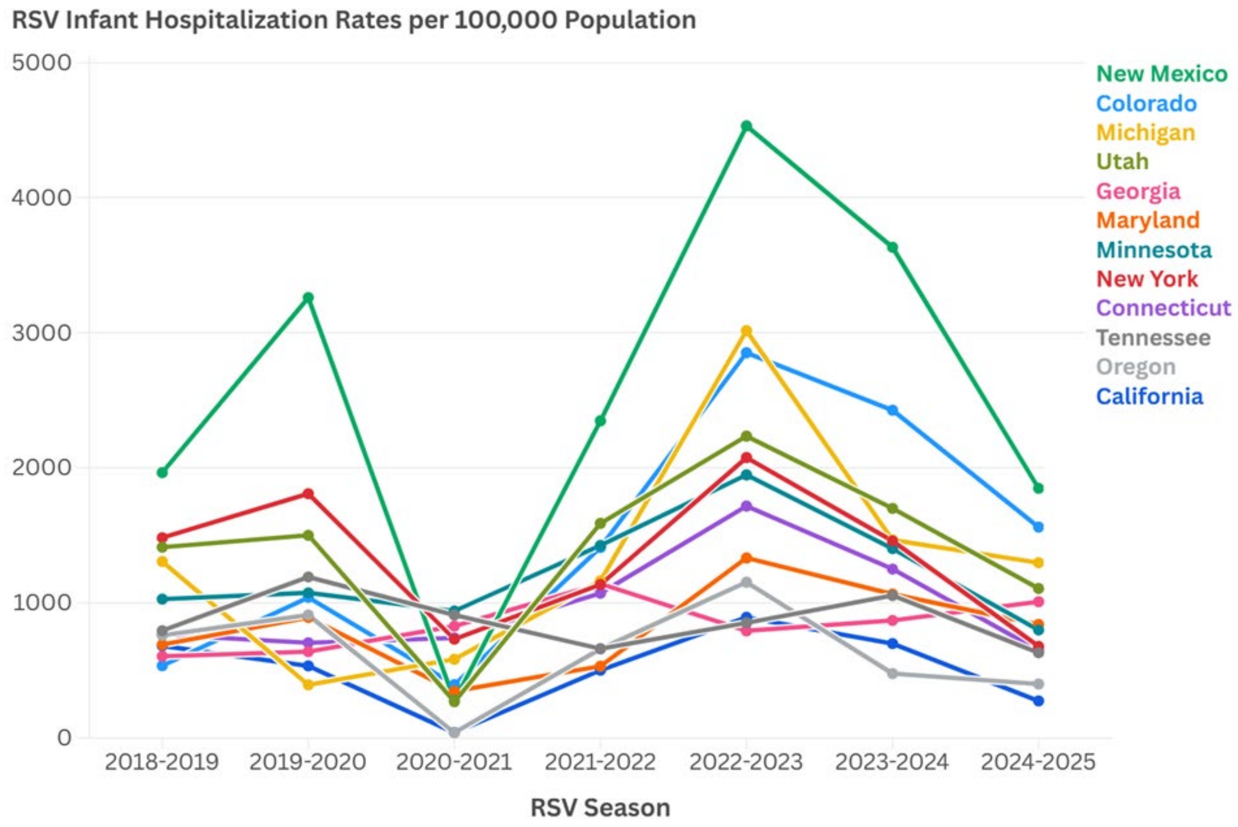

Note: Data for 2018–19 and 2019–20 RSV seasons were compiled only from October–April, representing typical RSV seasons before the COVID-19 pandemic. From 2020 season onward, data was collected for all months (October to September in the following year). Hospitalization rates are calculated as the number of infants in a surveillance area who are hospitalized with laboratory-confirmed RSV divided by the total age specific population estimate for that area.
